# Hexose Kinases and Their Role in Sugar-Sensing and Plant Development

**DOI:** 10.3389/fpls.2013.00044

**Published:** 2013-03-12

**Authors:** David Granot, Rakefet David-Schwartz, Gilor Kelly

**Affiliations:** ^1^Institute of Plant Sciences, The Volcani Center, Agricultural Research OrganizationBet Dagan, Israel

**Keywords:** hexokinase, fructokinase, glucose, fructose, sugar-sensing, intracellular localization, hexose-phosphorylation

## Abstract

Hexose sugars, such as glucose and fructose produced in plants, are ubiquitous in most organisms and are the origin of most of the organic matter found in nature. To be utilized, hexose sugars must first be phosphorylated. The central role of hexose-phosphorylating enzymes has attracted the attention of many researchers, leading to novel discoveries. Only two families of enzymes capable of phosphorylating glucose and fructose have been identified in plants; hexokinases (HXKs), and fructokinases (FRKs). Intensive investigations of these two families in numerous plant species have yielded a wealth of knowledge regarding the genes number, enzymatic characterization, intracellular localization, and developmental and physiological roles of several HXKs and FRKs. The emerging picture indicates that HXK and FRK enzymes found at specific intracellular locations play distinct roles in plant metabolism and development. Individual HXKs were shown for the first time to be dual-function enzymes – sensing sugar levels independent of their catalytic activity and controlling gene expression and major developmental pathways, as well as hormonal interactions. FRK, on the other hand, seems to play a central metabolic role in vascular tissues, controlling the amounts of sugars allocated for vascular development. While a clearer picture of the roles of these two types of enzymes is emerging, many questions remain unsolved, such as the specific tissues and types of cells in which these enzymes function, the roles of individual HXK and FRK genes, and how these enzymes interact with hormones in the regulation of developmental processes. It is anticipated that ongoing efforts will broaden our knowledge of these important plant enzymes and their potential uses in the modification of plant traits.

## The Origin of Hexoses and Other Sugars Found in Source and Sink Plant Tissues and Their Intracellular Localization

**Figure 1:**
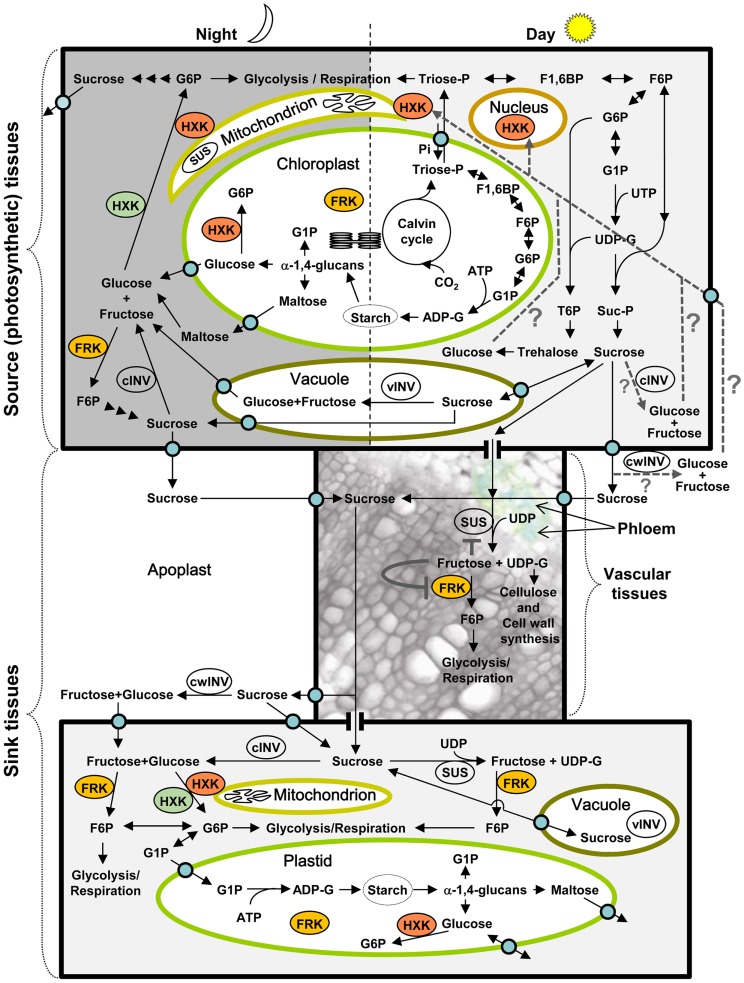
**Schematic presentation of sugar metabolism in source and sink tissues during the day and night, and localization of HXK and FRK enzymes in eudicots and monocots**. Triose-phosphate (Triose-P), the product of photosynthetic CO_2_ fixation in the Calvin cycle, is exported to the cytoplasm. Consecutive cytoplasmic enzymatic steps lead to the formation of G6P independent of HXK and FRK. Further metabolism of G6P yields sucrose, which remains in the cytosol, is temporarily stored in the vacuole or is exported to the apoplast. G6P metabolism may also yield T6P and trehalose. Within the chloroplast, Triose-P is used for the formation of starch during the day. During the dark period, starch is degraded to maltose, glucose, and glucose-1-phosphate (G1P). Maltose cleavage also releases glucose, and vacuolar and cytosolic sucrose might be cleaved by cytosolic (cINV) and vacuolar (vINV) invertases to produce glucose and fructose. While fructose can be phosphorylated by FRK, glucose must be phosphorylated by HXK. Orange stained HXK and FRK indicate the localization of these enzymes in monocots and eudicots. Cytosolic HXK (stained green) is also found in monocots. Sucrose transported to the apoplast, during the day or night enters the phloem via sucrose transporters. Within the vascular tissues, sucrose can be cleaved by sucrose synthase (SUS) to support vascular development or transported to other sink tissues. Sucrose unloading in sink tissues may proceed symplasmically via plasmodesmata or through the apoplast via sucrose transporters. Alternatively, sucrose might be cleaved by apoplastic (cell wall) invertase (cwINV) to produce glucose and fructose that would enter sink cells via specific hexose transporters. The enzymatic steps of sugar metabolism in sink tissues are similar to those found in source tissues. In addition to the metabolic function of HXK, this enzyme also senses the presence of glucose and represses the expression of photosynthetic genes in the nucleus of source photosynthetic tissues. The origin of the glucose in photosynthetic tissues that is sensed by HXK is not known. Potential sources are presented by dashed lines: cleavage of sucrose or trehalose within the cytosol, or apoplastic cleavage of sucrose followed by uptake of the released hexoses. The presumed role of fructose and FRK in vascular tissues is indicated by the gray lines. Some of the sucrose transported in the phloem is cleaved by SUS to support cellulose and cell wall synthesis and vascular development. The released fructose is phosphorylated by FRK, but if SUS cleaves too much sucrose, the concentration of fructose will increase and inhibit both SUS and FRK activity, thereby affecting the amount of sucrose allocated for vascular development. ADP, adenosine diphosphate; ADP-G, ADP-glucose; cINV, cytosolic invertase; cwINV, cell wall invertase; F1,6BP, fructose 1,6-biphosphate; F6P, fructose 6-phosphate; FRK, fructokinase; G6P, glucose 6-phosphate; G1P, glucose 1-phosphate; HXK, hexokinase; Suc-P, sucrose-phosphate; SUS, sucrose synthase; T6P, trehalose 6-phosphate; Triose-P, triose-phosphate; UDP, uridine diphosphate; UDP-G, UDP-glucose; vINV, vacuolar invertase. Blue circles represent transporters.

Sugars such as the disaccharide sucrose and the hexoses glucose and fructose are the primary products of photosynthesis and the initial building blocks of most natural organic matter. In photosynthesis, atmospheric carbon dioxide (CO_2_) is incorporated into organic molecules via the Calvin cycle in the chloroplast to yield triose-phosphates (Triose-P) (Figure 1). These Triose-P may then be exported to the cytoplasm, where two molecules of Triose-P are combined to form fructose 1,6-biphosphate (F1,6BP), the first phosphorylated hexose (hexose-P); that very same enzymatic reaction may take place in the chloroplast, as well. Following additional consecutive enzymatic reactions in the cytoplasm or chloroplast, F,16BP can yield fructose 6-phosphate (F6P), glucose 6-phosphate (G6P), glucose 1-phosphate (G1P), and the nucleotide sugars UDP-glucose (UDP-G), and ADP-glucose (ADP-G) (Figure 1) (Dennis and Blakeley, 2000).

In the chloroplast, ADP-G is used for starch synthesis, to store extra sugar and to form a pool of reserve carbohydrates. The starch stored in the chloroplast can be degraded into the disaccharide [α(1 → 4) glucose–glucose] maltose, glucose, or G1P, but probably only glucose and maltose are exported (via specific carriers) to the cytoplasm (Zeeman et al., 2007). The breakdown of maltose in the cytoplasm yields glucose monomers. Glucose must be phosphorylated to produce G6P, which can then be used for metabolic processes. G6P can be isomerized into F6P to initiate glycolysis, respiration, and catabolic and anabolic processes. Alternatively, it may be mutated to G1P and the phosphate (P) group may be replaced by nucleotide diphosphates, such as UDP and ADP, to form the nucleotide sugars UDP-G and ADP-G, which are substrates for many biosynthetic and glycosylation reactions (Bar-Peled and O’Neill, 2011). UDP-G may be combined with F6P to form sucrose-phosphate (Suc-P), which is dephosphorylated to produce sucrose. This sucrose can then be stored in the vacuole or exported out of the photosynthetic (source) tissues through the phloem to non-photosynthetic (sink) tissues, where it serves as an initial substrate for all organic metabolic pathways (Figure 1). UDP-G may also be combined with G6P to form trehalose-6-phosphate (T6P), which is dephosphorylated to produce trehalose. T6P and trehalose usually exist in very small amounts in plants, coordinating metabolism with plant growth (Paul et al., 2008).

Sucrose, a glucose-fructose disaccharide, is the main photoassimilate transported from source to sink tissues in many plants. Some plant families, such as the Cucurbitaceae, also translocate raffinose-family oligosaccharides (RFOs), which are galactosyl derivatives of sucrose that contain one or more galactose moieties (Keller and Pharr, 1996). However, the metabolism of the RFOs in sink tissues starts with the removal of the galactose moieties and the release of sucrose (Gao and Schaffer, 1999; Carmi et al., 2003). A few plant species translocate other sugars, such as the sugar alcohols sorbitol or mannitol, or mannoheptulose. Nevertheless, those species also produce and translocate sucrose and the translocated alcohols are eventually converted into fructose in sink tissues (Rennie and Turgeon, 2009; Turgeon and Wolf, 2009). Hence, sucrose and hexose metabolism are probably ubiquitous in all plant species.

Upon arriving in sink tissues, sucrose may be metabolized immediately, stored in vacuoles or converted into and stored as starch in plastids, following several enzymatic reactions. In both sink and photosynthetic source tissues, to be metabolized, sucrose must be cleaved by either invertase (INV) or sucrose synthase (SUS), the only two families of sucrose-cleaving enzyme that have been identified in plants (Dennis and Blakeley, 2000). INV cleaves sucrose into the monomer hexoses glucose and fructose; whereas SUS cleaves sucrose in the presence of UDP to yield fructose and UDP-G. It has been suggested that SUS may also cleave sucrose in the presence of ADP instead of UDP, to yield fructose and ADP-G (Baroja-Fernandez et al., 2003; Munoz et al., 2005). ADP-G may be used for starch biosynthesis; whereas UDP-G can be used in various metabolic processes, such as cellulose synthesis, re-synthesis of Suc-P, and glycosylation reactions (Dennis and Blakeley, 2000; Munoz et al., 2005; Bar-Peled and O’Neill, 2011). However, the hexoses glucose and fructose must be phosphorylated before they can be used in metabolic processes. While SUS has been localized in the cytoplasm and in mitochondria and also appears to be associated with the plasma membrane and Golgi membrane, INVs are located in the apoplast (cell wall INV – cwINV), cytosol (cytoplasmic invertase-cINV), and vacuoles (vINV) (Amor et al., 1995; Carlson and Chourey, 1996; Buckeridge et al., 1999; Dennis and Blakeley, 2000; Winter and Huber, 2000; Roitsch and Gonzalez, 2004; Subbaiah et al., 2006) (Figure 1). The degradation of starch releases plastidic and cytoplasmic glucose, and glucose may also enter plastids via a plastidic glucose transporter (Weber et al., 2000; Butowt et al., 2003). Hence, glucose and fructose may be present in the apoplast, cytoplasm, vacuoles, and plastids. The apoplastic glucose and fructose must enter the cells to be phosphorylated, as there is no evidence for any extracellular hexose-phosphorylating enzymes.

## Discovery of HXKs and FRKs and Their Substrates

To date, only two types of glucose- and fructose-phosphorylating enzymes have been discovered in plants, hexokinases (HXKs), and fructokinases (FRKs). HXKs and FRKs were first purified from protein extracts of sink and source tissues by ion-exchange chromatography in the early 1950s (Millerd et al., 1951; Saltman, 1953; Medina and Sols, 1956). Several HXK and FRK isozymes have been identified in protein extracts of various plant species. While FRK activities have been found to be specific to fructose, the HXKs from various species have been found to be capable of phosphorylating glucose, fructose, mannose, and glucosamine, but not galactose (Saltman, 1953), similar to fungal, mammalian, and protozoan HXKs (Otieno et al., 1975; Cardenas et al., 1984; Doehlert, 1989, 1990; Xu et al., 1995; Panneman et al., 1998; Kroschewski et al., 2000; Fekete et al., 2004; Rui and Hahn, 2007). Isozymes capable of phosphorylating glucose have often been identified as glucokinases. However, unlike the situation in the fungal kingdom, no glucose-specific glucokinase has been found in plants (Dai et al., 2002b). Hence, in plants, glucose can be phosphorylated only by HXK while fructose can be phosphorylated by either HXK or FRK. As such, HXK and FRK are the gateway for most organic metabolism in plants. These enzymes catalyze irreversible reactions and therefore may play important roles in the regulation of plant sugar metabolism.

## Occurrence of *HXK* and *FRK* Genes in Various Plant Species

The potato (*Solanum tuberosum*) FRK (*StFRK*) was the first hexose-phosphorylating gene to be isolated from a plant (Smith et al., 1993; Taylor et al., 1995) and the first HXK gene was isolated from Arabidopsis (*Arabidopsis thaliana*, *AtHXK1*) (Dai et al., 1995). Since those first discoveries, several HXK and FRK genes have been isolated from different plant species (Tables 1 and 2). For example, four HXK and four FRK genes have been isolated from tomato (*Solanum lycopersicum*), three HXK and three HXK-like (HKL) genes have been isolated from Arabidopsis (*Arabidopsis thaliana*) and 10 HXK genes have been isolated from rice (*Oryza sativa*) (Dai et al., 2002a; German et al., 2002, 2004; Cho et al., 2006a; Guglielminetti et al., 2006; Karve et al., 2008, 2010). Several other FRK isozymes from a few different species have been isolated and characterized and there are also examples of FRKs that have been identified based only on their partial protein sequence, though the genes corresponding to these enzymes have not yet been identified (Table 2).

**Table 1:** **Hexokinase genes and their physiological function**.

Species	Gene	Accession no	Type/intracellular localization	Physiological function	Reference
**EUDICOTS**					
*Arabidopsis thaliana*	*AtHXK1*	AT4G29130	Type B/M, N	Glc sensing, PCD, mediates sugar and hormonal interactions, growth and development, photosynthetic gene repression, transpiration, actin-filament reorganization, oxidative-stress response, pathogen resistance, directional root growth, leaf senescence	Jang et al. (1997), Dai et al. (1999), Yanagisawa et al. (2003), Moore et al. (2003), Leon and Sheen (2003), Kim et al. (2006), Pourtau et al. (2006), Cho et al. (2006a), Rolland et al. (2006), Chen (2007), Aki et al. (2007), Balasubramanian et al. (2007, 2008), Sarowar et al. (2008), Karve et al. (2008), Ju et al. (2009), Karve et al. (2010), Kushwah et al. (2011), Kelly et al. (2012)
	*AtHXK2*	AT2G19860	Type B/M	Glc sensing, PCD, photosynthetic gene repression	Jang et al. (1997), Kim et al. (2006), Karve et al. (2008)
	*AtHXK3*	AT1G47840	Type A/P	Glc sensing, abiotic stress response	Claeyssen and Rivoal (2007), Karve et al. (2008), Zhang et al. (2010)
	*AtHKL1*	AT1G50460	Type B/M	Growth, root hair development, mediates Glc-ethylene crosstalk, abiotic stress response	Claeyssen and Rivoal (2007), Karve et al. (2008), Karve and Moore (2009), Karve et al. (2012)
	*AtHKL2*	AT3G20040	Type B/M		Karve et al. (2008)
	*AtHKL3*	AT4G37840	Type B/M	Abiotic stress response	Claeyssen and Rivoal (2007), Karve et al. (2008)

Tomato (*Solanum lycopersicum*)	*SlHXK1*	AJ401153	Type B/M		Damari-Weissler et al. (2006)
	*SlHXK*2	AF208543	Type B/M		Menu et al. (2001), Damari-Weissler et al. (2006)
	*SlHXK3*	DQ056861	Type B/M		Kandel-Kfir et al. (2006)
	*SlHXK4*	DQ056862	Type A/P		Kandel-Kfir et al. (2006)

*Solanum chacoense*	*ScHK2*	DQ177440	ND		Claeyssen et al. (2006)

Potato (*Solanum tuberosum*)	*StHXK1*	X94302	ND	Glc sensing, Leaves starch content,	Veramendi et al. (1999), Veramendi et al. (2002)
	*StHXK2*	AF106068	ND	Glc sensing	Veramendi et al. (2002)

Tobacco (*Nicotiana tabacum/benthamiana*)	*NtHXK2*	AY553215	Type A/P		Giese et al. (2005)
	*NbHXK1*	AY286011	Type B/M	Plant growth, PCD, oxidative-stress resistance	Kim et al. (2006), Sarowar et al. (2008)

Sunflower (*Helianthus annuus*)	*HaHXK1*	DQ835563	ND	Seed development	Troncoso-Ponce et al. (2011)

Poplar (*Populus trichocarpa*)	*PtHXK1*	XP_002325031	Type B/M	Glc sensing	Karve et al. (2010)
Grape (*Vitis vinifera* L. cv. Cabernet Sauvignon)	*VvHXK1*	JN118544	ND		Yu et al. (2012)
	*VvHXK2*	JN118545	ND		Yu et al. (2012)

Spinach (*Spinacia oleracea*)	*SoHXK1*	AF118132	Type B/M		Wiese et al. (1999), Damari-Weissler et al. (2007)

**MONOCOTS**					
Rice *(Oryza sativa)*	*OsHXK1*	DQ116383	Type C/C, N		Cho et al. (2006a), Cheng et al. (2011)
	*OsHXK2*	DQ116384	Type B/M		Cheng et al. (2011)
	*OsHXK3*	DQ116385	Type B/M		Cheng et al. (2011)
	*OsHXK4*	DQ116386	Type A/P		Cho et al. (2006a), Cheng et al. (2011)
	*OsHXK5*	DQ116387	Type B/M, N	Glc sensing, photosynthetic gene repression, Shoot growth	Cho et al. (2009a), Cheng et al. (2011)
	*OsHXK6*	DQ116388	Type B/M, N	Glc sensing, photosynthetic gene repression, Shoot growth	Aki and Yanagisawa (2009), Cho et al. (2009a), Cheng et al. (2011)
	*OsHXK7*	DQ116389	Type C/C, N		Cho et al. (2006a), Cheng et al. (2011)
	*OsHXK8*	DQ116390	Type C/C, N		Cheng et al. (2011)
	*OsHXK9*	DQ116391	Type B/M		Cheng et al. (2011)
	*OsHXK10*	DQ116392	C and/or M	Pollen germination	Xu et al. (2008), Cheng et al. (2011)

Sorghum (*Sorghum bicolor*)	*SbHXK3*	XP_002459072	Type B/M	No Glc sensing role	Karve et al. (2010)
	*SbHXK8*	XP_002455027	C		Karve et al. (2010)

Wheat *(Triticum aestivum)*	HXK	AY974231	ND	Controls triose-phosphate/phosphate translocation	Sun et al. (2006)
**LYCOPHYTES**					

Spike moss (*Selaginella mollendorffii*)	*SmHXK3*	26000047*	C	Glc sensing	Karve et al. (2010)
	*SmHXK5*	57.357.1*	C		Karve et al. (2010)

**BRYOPHYTES**					
Moss (*Physcomitrella patens)*	*PpHXK1*	AY260967	Type A/P	Filamentous type and growth	Olsson et al. (2003), Thelander et al. (2005)
	*PpHXK2*	XM_001784578	Type B/M, P		Nilsson et al. (2011)
	*PpHXK3*	XM_001784282	Type B/M, P		Nilsson et al. (2011)
	*PpHXK4*	XM_001760896	Type C/C, N		Nilsson et al. (2011)
	*PpHXK5*	XM_001766381	Type A/P		Nilsson et al. (2011)
	*PpHXK6*	XM_001762899	Type A/P		Nilsson et al. (2011)
	*PpHXK7*	XM_001754096	Type B/M, P		Nilsson et al. (2011)
	*PpHXK8*	XM_001752177	Type B/M, P		Nilsson et al. (2011)
	*PpHXK9*	XM_001770125	Type D/M		Nilsson et al. (2011)
	*PpHXK10*	XM_001776713	Type D/M		Nilsson et al. (2011)
	*PpHXK11*	XM_001779426	Type D/M, P		Nilsson et al. (2011)

*Type A, localized in plastid stroma; Type B, associated with the mitochondria; Type C, localized in the cytosol and nucleus; Type D, associated with the mitochondria, different from type B in sequence; M, mitochondria-associated; P, plastid; N, nucleus; C, cytosol; ND, not determined; PCD, programmed cell death; Glc, glucose*.

*Joint Genome Institute- *Selaginella moellendorffii* v1.0 database (http://genome.jgi-psf.org/Selmo1/Selmo1.home.html).

**Table 2:** **Fructokinase genes and isozymes and their physiological functions**.

Species	Gene/Isozyme	Accession no	Substrate inhibition	Intracellular localization	Physiological function	Reference
**EUDICOTS**						
Pea (*Pisum sativum*)	FRK2	NA	Yes	ND		Copeland et al. (1984)
	FRK1	NA	Yes	ND		Turner et al. (1977)

Soybean (*Glycine max*)	FRK	NA	Yes	C		Copeland and Morell (1985a,b)

Honey locust (*Gleditsia triacanthos*)	FRK	NA	Yes	ND		Myers and Matheson (1994)

*Arabidopsis thaliana*	FRK1	NA	Yes	ND		Gonzali et al. (2001)
	FRK2	NA	Yes	ND		Gonzali et al. (2001)

Tomato (*Solanum lycopersicum*)	*SlFRK1*/FKII	U64817/AAB57733	No	C	Promotes transition to flowering	Kanayama et al. (1997), Kanayama et al. (1998). Petreikov et al. (2001), Odanaka et al. (2002)
	*SlFRK2*/FKI	U64818/AAB57734	Yes	C	Stem and root growth xylem and seed development	Kanayama et al. (1997), Kanayama et al. (1998), Petreikov et al. (2001), Odanaka et al. (2002), Dai et al. (2002a), German et al. (2003)
	*SlFRK3/*FKIII	AY323226/AAR24912, Q6VWJ5	Yes	P		Petreikov et al. (2001), German et al. (2004)
	*SlFRK4*	AY099454/AAM44084	No	C	*Involved in pollen development	German et al. (2002), David-Schwartz et al. (2013)

Potato (*Solanum tuberosum*)	*StFK1*/FRK	Z12823/CAA78283	Yes	ND	Regulates sucrose metabolism together with SUS	Smith et al. (1993), Taylor et al. (1995), Dai et al. (1997), Davies et al. (2005)
	FK1	NA	Yes	ND		Gardner et al. (1992)
	FK2	NA	Yes	ND		Gardner et al. (1992)
	FK3	NA	No	ND		Gardner et al. (1992)
	FK	NA	ND	ND	*Expressed in vascular tissue	Sergeeva and Vreugdenhil (2002)

Sunflower (*Helianthus annuus*)	FRK3	NA	ND	ND	*Involved in response to drought stress	Fulda et al. (2011)

Camellia (*Camellia japonica*)	FRK	NA	Yes	ND	*Involved in pollen development and function	Nakamura et al. (1991)
Spinach (*Spinacia oleracea*)	FK I	NA	Yes	C		Schnarrenberger (1990)
	FK II	NA	No	P		Schnarrenberger (1990)

Sugar beet (*Beta vulgaris*)	FK	NA	Yes	ND	*Associated with vascular development	Chaubron et al. (1995)

**MONOCOTS**						
Barley (*Hordeum vulgare*)	FK Ia	NA	Yes	ND		Baysdorfer et al. (1989)
	FK Ib	NA	Yes	ND		Baysdorfer et al. (1989)
	FK II	NA	No	ND		Baysdorfer et al. (1989)

Rice (*Oryza sativa*)	*OsFKI*/OsFKI	AF429948/AAL26573	Yes	ND	*Dominant under aerobic conditions, up-regulated in late stages of pollen development, accumulates in grains	Jiang et al. (2003), Kerim et al. (2003), Guglielminetti et al. (2006)
	*OsFKII*/OsFKII	AF429947/AAL26574	No	ND	*Involved in response to anoxic conditions	Jiang et al. (2003), Guglielminetti et al. (2006)
	FK	NA	ND	ND	*Supports the glycolytic pathway for ATP production under anoxic conditions	Kato-Noguchi (2004)

Maize (*Zea mays*)	FK-1	NA	Yes	ND		Doehlert (1989, 1990)
	FK-2	NA	Yes	ND		
	*ZmFRK1*/ZmFrk1	AY197772/AAP42805	Yes	ND	*Cellulose synthesis	Zhang et al. (2003), Melida et al. (2011)
	*ZmFRK2*/ZmFrk2	AY197773/AAP42806	Yes	ND	*Involved in response to salt stress	Zhang et al. (2003), Zorb et al. (2011)

Sugarcane (*Saccharum officinarum*)	FRK1	NA	No	ND		Hoepfner and Botha (2004)
	FRK2	NA	Yes	ND		Hoepfner and Botha (2004)
Lily (*Lilium longiflorum* and *L. lancifolium*)	FRK	NA	Yes	ND	*Pollen development and function	Nakamura et al. (1991)

*Predicted physiological function through the examination of gene expression profiles.

*NA, not available; ND, not determined; C, cytosol; P, plastid*.

The increased availability of plant genome sequences allowed Karve et al. (2010) to estimate the number of HXK genes in the moss *Physcomitrella patens*, the lycophyte *Sellaginella mollendorffii*, three eudicot species, and three monocot species. They concluded that the number of sequences associated with HXK gene families ranged from 11 sequences in *Physcomitrella* to 5–7 sequences for *Sellaginella* and the eudicot species, and 8–10 sequences in the monocot species. Based on genome sequence data, Thelander et al. (2009) estimated that there are seven FRK genes in Arabidopsis, three in rice, and eight in *Physcomitrella*. It appears that both HXK and FRK exist in species from the main land plant lineages, including mosses, lycophytes, gymnosperms, and angiosperms (Tables 1 and 2).

## Enzymatic Characterization of *HXK*- and *FRK*-Encoded Isozymes

The enzymatic activity and biochemical characteristics of a limited number of enzymes encoded by isolated *HXK* and *FRK* genes were determined following the expression of those genes in yeast, bacteria, or plant protoplasts. These genes were isolated from Arabidopsis (three HXK and three HXK-like genes), tomato (four HXK and four FRK genes), potato (a single FRK gene), *Solanum chacoense* (a wild relative of the cultivated potato), sunflower (*Helianthus annuus*; a single HXK gene), *Sorghum bicolor*, grape (*Vitis vinifera*; two HXK genes), rice (10 genes), and *Physcomitrella* and *Sellaginella* (three HXK genes each) (Taylor et al., 1995; Dai et al., 1997, 1999, 2002b; Kanayama et al., 1997, 1998; Menu et al., 2001; Petreikov et al., 2001; German et al., 2002, 2004; Cho et al., 2006a; Claeyssen et al., 2006; Kandel-Kfir et al., 2006; Karve et al., 2010; Nilsson et al., 2011; Troncoso-Ponce et al., 2011; Yu et al., 2012). Unlike the Arabidopsis HXK-like (HKL) genes, the three Arabidopsis HXKs do exhibit hexose-phosphorylation catalytic activity (Karve et al., 2008). A few *HXK* genes from *Sorghum* and *Sellaginella* also lack glucose phosphorylation catalytic activity, suggesting that *HKL* genes might be present in various species (Karve et al., 2010). Biochemical characterization of the Arabidopsis, tomato, potato, sunflower, and grape enzymes encoded by the isolated genes revealed that the affinity of HXK for glucose is in the 0.02–0.1 mM range; whereas its affinity for fructose is about one to three orders of magnitude lower, in the 2–120 mM range (Dai et al., 1999; Claeyssen et al., 2006; Granot, 2007; Moisan and Rivoal, 2011; Troncoso-Ponce et al., 2011; Yu et al., 2012). The affinity of FRK for fructose is usually high within the same range as the affinity of HXK for glucose (Taylor et al., 1995; Pego and Smeekens, 2000; Granot, 2007), though the affinity of the tomato SlFRK1 for fructose is relatively low (1.3 mM) (German et al., 2004). Since the affinity of HXK for glucose is much higher than its affinity for fructose, it has been suggested that *in vivo* HXK probably phosphorylates mainly glucose; whereas FRK might mainly phosphorylate fructose (Granot, 2007). However, the intracellular location of these enzymes and their substrates may also affect which enzyme phosphorylates which hexose (see Intracellular Localization of HXKs and FRKs).

An interesting phenomenon has been noted for several FRK isozymes. Unlike the usual Michaelis–Menten kinetics of increased activity with increased substrate concentration, the potato StFRK, and the tomato SlFRK2 and SlFRK3 enzymes exhibit substrate inhibition, a phenomenon first observed with FRKs from pea (*Pisum sativum*) (Turner et al., 1977; Renz and Stitt, 1993; Dai et al., 1997; Martinez-Barajas et al., 1997). Namely, these enzymes are inhibited by their own substrate, fructose, when its concentration exceeds a certain level, usually about 0.5–1 mM (Turner et al., 1977; Dai et al., 1997; Petreikov et al., 2001; German et al., 2004). The Arabidopsis FRK1 and FRK2 isozymes exhibit substrate inhibition at fructose concentrations above 5 mM (Gonzali et al., 2001). Substrate inhibition has been reported for FRKs from additional species, including monocots and eudicots (Pego and Smeekens, 2000; Table 2). Sucrose synthase (SUS), which cleaves sucrose to yield UDP-G and fructose, is also inhibited by similar concentrations of fructose (Schaffer and Petreikov, 1997a). Therefore, it has been postulated that FRK substrate inhibition has evolved to impose a “double-brake” mechanism to prevent excess cleavage and consumption of sucrose in various tissues, such as potato tubers (Pego and Smeekens, 2000). It has also been suggested that the inhibition of both enzymes by fructose may cause a shift in the equilibrium of SUS-catalyzed reaction toward sucrose synthesis (Renz and Stitt, 1993). The biological significance of such a mechanism in tissues like potato tubers that accumulate starch is not yet clear (Davies et al., 2005), but it has been proposed that such a mechanism might control the amount of sucrose used for vascular development (German et al., 2003; Granot, 2007).

## Intracellular Localization of HXKs and FRKs

The intracellular localization of HXK and FRK isozymes has been the subject of intense examination. Initial HXK and FRK localization studies using cellular fractionation methods have been carried out in a variety of plant species, including mung bean, potato, wheat, and several others (Copeland and Morell, 1985a; Copeland and Tanner, 1988; Schnarrenberger, 1990). Most of these studies suggested that *in vivo* the majority of HXK activity is associated with the mitochondria, while there is also some HXK activity associated with plastids. A few studies suggested that HXK may be present in the cytosol as well (reviewed in Granot, 2008; Troncoso-Ponce et al., 2009). FRK activity was usually identified in the cytosolic fraction (Copeland and Morell, 1985a; Troncoso-Ponce et al., 2009). However, the major breakthrough concerning the intracellular localization of plant HXKs and FRKs was achieved with the cloning of HXK and FRK genes. With the sequences of HXK and FRK genes in hand, it became possible to look for signal peptides that may indicate the intracellular localization of specific HXK and FRK isozymes and to verify that localization using tagged proteins (Tables 1 and 2).

### HXK localization

Based on their N-terminal amino acid sequences, plant HXK genes were first classified into two major groups, type A and type B (Olsson et al., 2003). Type A HXKs have a chloroplast transit peptide of about 30 amino acids; whereas type B HXKs share a common N-terminal hydrophobic membrane anchor domain of about 24 amino acids and are probably associated with membranes. Studies with GFP fusion proteins have localized type A HXK isozymes of moss (*P. patens*), tobacco (*Nicotiana tabacum*), tomato, rice, and Arabidopsis within plastid stroma (Olsson et al., 2003; Giese et al., 2005; Cho et al., 2006a; Kandel-Kfir et al., 2006; Karve et al., 2008). The tomato plastidic HXK (SlHXK4) has also been observed within stromules, which are stroma-filled tubular extensions of the plastid envelope that form connections between plastids to allow the transport of proteins between plastids (Kohler et al., 1997; Kandel-Kfir et al., 2006).

The intracellular locations of type B HXK isozymes have been determined by several means. Proteomic analysis of mitochondrial proteins has located Arabidopsis AtHXK1 and AtHXK2 enzymes on the outside of the mitochondrial membrane (Giege et al., 2003; Heazlewood et al., 2004). Studies based on the use of GFP fusion proteins have shown that Arabidopsis AtHXK1 and AtHXK2, tomato SlHXK1, 2, and 3, *Nicotiana benthamiana* NbHXK1, rice OsHXK 2, 3, 5, 6, and 9, *Sorghum* SbHXK3, *Populus trichocarpa* PtHXK1, and spinach (*Spinacia oleracea*) SoHXK1 are associated with mitochondria (Cho et al., 2006a; Damari-Weissler et al., 2006, 2007; Kandel-Kfir et al., 2006; Kim et al., 2006; Balasubramanian et al., 2007; Karve et al., 2008, 2010; Cheng et al., 2011). Deletion of the putative N-terminal membrane anchor domains of the Arabidopsis AtHXK1, tomato SlHXK 1, 2, and 3, and the *N. benthamiana* NbHXK1 proteins resulted in their localization to the cytosol, confirming that this domain is a membrane anchor domain (Damari-Weissler et al., 2006; Kim et al., 2006; Balasubramanian et al., 2007). Lastly, fusion of the Arabidopsis AtHXK1 N-terminal domain to GFP was sufficient to cause mitochondrial association (Balasubramanian et al., 2007), indicating that the N-terminal membrane anchor domain of a type B HXK determines mitochondrial association. None of the HXK isozymes deduced from the numerous HXK genes that have been cloned from seed plants seem to be located on the outer plastidic envelope or on the plasma membrane, as had been suggested previously (Stitt et al., 1978; Wiese et al., 1999; Claeyssen et al., 2006; Claeyssen and Rivoal, 2007; Granot, 2008). The type B HXKs of Arabidopsis (AtHXK1) and rice (OsHXK5 and OsHXK6) were also found in the nucleus, where they might regulate gene expression (Cho et al., 2006b, 2009a).

To date, all of the HXKs examined in eudicots have been found to have either a plastidic signal peptide (type A) or an N-terminal membrane anchor domain (type B) (Olsson et al., 2003; Claeyssen et al., 2006; Granot, 2007). However, cytosolic HXK were also identified in monocots. Four of the 10 rice HXKs, OsHXK1, OsHXK7, OsHXK8, and OsHXK10 lack or have a truncated N-terminal membrane anchor domain and are located in the cytosol (Cho et al., 2006a; Cheng et al., 2011). Cheng et al. (2011) suggested that OsHXK1, OsHXK7, and OsHXK8 are also located within nuclei.

Karve et al. (2010) developed a way to predict the localization of HXK based on sequence identity. Their prediction analysis supports the three locations in seed plants mentioned above: in association with the mitochondria, the plastid stroma, and the cytosol (in monocots). HXK were also found in plastids, associated with the mitochondria and in the cytosol of the primitive plant species *Physcomitrella*, and in the cytosol and associated with the mitochondria of the lycophyte *Selaginella* (Karve et al., 2010; Nilsson et al., 2011). Nilsson et al. (2011) suggested that in addition to types A and B, *Physcomitrella* has two new types of HXK with no obvious orthologs in vascular plants. Type C, encoded by a single gene, has neither transit peptide nor membrane anchor, and is found in the cytosol and in the nucleus. Type D HXKs, encoded by three genes, have membrane anchors and are associated with the mitochondria, but their sequences differ from those of the type B HXK. One type D HXK, *PpHXK1*, is also found on the chloroplast envelope (Nilsson et al., 2011). Interestingly, the 11 putative *Physcomitrella* HXKs are more closely related to each other than to any HXK from vascular plants. This is in contrast to the situation in seed plants, in which HXKs of the same type from different plants typically are more closely related to each other than HXKs of different types from the same plant (Karve et al., 2010; Nilsson et al., 2011). Nevertheless, the sequence of the membrane anchor domain in the type B HXK is highly conserved between seed plants and *Physcomitrella*. Nilsson et al. (2011) suggested that several genes encoding different types of HXK may have been present in the common ancestor of mosses and seed plants and that, unlike the situation in seed plants, these genes may have co-evolved in *Physcomitrella* by gene conversion, making them appear to be more closely related to each other than they really are (Nilsson et al., 2011).

### FRK localization

The intracellular localization of FRK isozymes has been studied with GFP fusion proteins of the four tomato FRKs (Damari-Weissler et al., 2006). Three of these tomato FRKs are located in the cytosol and one (SlFRK3) is located within plastids and stromules. Although previously suggested (Schnarrenberger, 1990; Singh et al., 1993; Wiese et al., 1999), the presence of FRK in plastids is quite surprising since, unlike glucose, the source of the fructose found in plastids is not clear. It is assumed that fructose could be transported into plastids by a fructose or hexose transporter or formed within plastids following the cleavage of sucrose. There is evidence that fructose may enter plastids through carrier-mediated diffusion (Schafer and Heber, 1977), but no fructose transporter has been characterized to date. Efficient movement of sucrose into plastids has been suggested (Gerrits et al., 2001) and a recently identified plastidic INV (Vargas et al., 2008) may also explain the presence of fructose in plastids. Nevertheless, the presence of FRK in plastids implies that fructose phosphorylation does occur in plastids.

The association of HXK with mitochondria and the cytosolic localization of FRK in eudicots may suggest that, in the cytoplasm, glucose is phosphorylated adjacent to the mitochondria; whereas fructose might be phosphorylated either adjacent to the mitochondria by HXK or in the cytosol by cytoplasmic FRKs. Considering the higher affinity of FRKs for fructose, as compared to that of HXKs, it is possible that fructose phosphorylation is primarily carried out by FRK in the cytosol. Such an intracellular spatial separation of glucose and fructose phosphorylation may raise the as yet unexplored option of microcompartmentalization and routing of glucose and fructose within the cytoplasm toward the mitochondria-associated HXK and the cytosolic FRK, respectively.

## Expression Pattern of HXK and FRK

Theoretical considerations may predict temporal and spatial expression patterns of HXKs and FRKs in different plant organs and tissues. For example, there is presumably no need for HXK and FRK in photosynthetic source tissues during the day as there is seemingly no production of free glucose or fructose in those tissues in the presence of light (Figure 1). Rather, in photosynthetic (source) tissues, HXK and FRK might be needed *a priori* mainly during the dark period, when starch and sucrose degradation yield glucose and fructose monomers. In contrast, in sink (non-photosynthetic) tissues, HXK and FRK might be required during both dark and light periods. Nevertheless, temporal and spatial expression patterns of HXKs and FRKs do not necessarily conform to these theoretical expectations. It appears that HXK and FRK genes are expressed in both source and sink tissues regardless of the time of day (Jang and Sheen, 1997; German et al., 2004; Cho et al., 2006a; Kandel-Kfir et al., 2006). Yet, in most cases, it is not known in which specific tissues and cell types these genes are expressed.

Almost all of the studied HXK and FRK genes are expressed at various levels in almost all plant organs. One exception is the cytosolic tomato *SlFRK4*, which is expressed specifically in anthers and pollen (German et al., 2002). This specific expression has been confirmed with the GUS reporter gene expressed under the *SlFRK4* promoter (David-Schwartz et al., 2013). FRKs that are specifically expressed in anthers and pollen are apparently present in other plants species as well, but the reason for a specific FRK in these organs is not known (David-Schwartz et al., 2013). The other three tomato FRK genes, *SlFRK1*, *2*, and *3*, seem to be expressed primarily in vascular tissues (unpublished data). Another exception is the tobacco plastidic *HXK2*. Promoter expression analysis of the tobacco *HXK2*, in which GUS was used as a reporter gene, indicated that it is expressed mainly in cells of the vascular starch sheath and xylem parenchyma, guard cells and root tips (Giese et al., 2005).

## Physiological Roles of HXK and FRK

It has been proposed that sugar levels and metabolism in the plant are monitored and regulated to ensure optimal growth. Due to their central roles in sugar metabolism, HXK, and FRK are promising candidates for the role of coordinating sugar metabolism with plant development and physiology.

### Role of HXK

The physiological roles of HXK genes have been studied in several different ways: through the exposure of plant-cell cultures, seeds, seedlings, and plants to exogenous sugar and sugar analogs; through the modification of the expression of HXK in transgenic plants and through the selection and analysis of mutants. The effects of each of these treatments on gene expression, plant physiology, and plant development have been observed.

In the early 1990s, it was first suggested that, in addition to their metabolic function, plant HXK might play a sugar-sensing role in the regulation of sugar metabolism, similar to that of yeast HXK, which controls sugar metabolism (Gancedo, 1992; Jang and Sheen, 1994). To study this hypothesis, maize protoplasts were exposed to exogenous sugars and the effect of this exposure on photosynthetic gene expression was monitored (Jang and Sheen, 1994). Repressed expression of photosynthetic genes such as rubisco (RBCS) and chlorophyll A/B binding protein (CAB) were accepted as markers for the study of sugar-sensing in plants (Jang and Sheen, 1997; Jang et al., 1997; Moore et al., 2003). Sugars that are substrates of HXK repressed the expression of these genes; whereas sugar analogs that are not substrates of HXK had no effect. Furthermore, the glucose analog 2-deoxyglucose, which is phosphorylated by HXK, but is not further metabolized, also inhibited the expression of the photosynthetic genes (Jang and Sheen, 1994). Similar results were also obtained in a study of Arabidopsis seedlings grown on synthetic media in the presence of glucose and glucose analogs (Jang and Sheen, 1997). These results established the notion that, in addition to its metabolic function, HXK plays a sugar-sensing role independent of the downstream metabolism of its products G6P and F6P (Jang and Sheen, 1997; Jang et al., 1997). This dual-function of HXK was confirmed when the catalytic and the signaling activities of Arabidopsis HXK1 (*AtHXK1*) were uncoupled (Moore et al., 2003). Two catalytically inactive HXK1 alleles with an amino acid substitution in their catalytic domains (G104D and S177A) mediated glucose repression of chlorophyll accumulation and photosynthetic gene expression in the *AtHXK1* loss-of-function *gin2* (*glucose insensitive 2*) mutant background (Moore et al., 2003). These results confirmed that *AtHXK1* is a dual-function enzyme that possesses both metabolic hexose-phosphorylation activity and a glucose-sensing function that is independent of its catalytic phosphorylation activity.

Repression of chlorophyll accumulation and photosynthetic gene expression by type B HXKs such as *AtHXK1* implies the presence of free glucose in the cytoplasm of photosynthetic tissues during the day. This is in contrast to the theoretical considerations described above, which questioned whether free glucose is present and whether HXK is needed in photosynthetically active mesophyll cells during the day. Yet, available glucose may originate from various sources in photosynthetically active mesophyll cells during the day, as depicted in Figure 1. This glucose may be generated from the degradation of starch, the cleavage of intracellular sucrose by cINV and/or the cleavage of some of the exported extracellular sucrose by cell wall invertase (cwINV) and subsequent importation. This glucose could also be generated through the cleavage of trehalose (a glucose–glucose disaccharide) by trehalase (Figure 1). The trehalose metabolic pathway has emerged as an important regulatory mechanism in plants, affecting sugar metabolism and plant growth (Paul et al., 2008). Trehalose cleavage as a source of glucose sensed by HXK might seem plausible as the involvement of a trehalose pathway in sensing sucrose level and sugar status of the cell has been suggested (Paul et al., 2008). Yet, the effects of trehalose on plant growth and sugar metabolism occur independently of the expression level of *AtHXK1*, perhaps eliminating trehalose as a potential source of the glucose sensed by HXK (Ramon et al., 2007).

Most studies of the signaling role of HXK in plants have involved the Arabidopsis *AtHXK1* gene, which encodes a mitochondria-associated (type B) enzyme (Rolland and Sheen, 2005). Overexpression of *AtHXK1* in Arabidopsis plants, under the control of the 35S promoter, inhibited seedling development, cotyledon greening and the expression of photosynthetic genes upon germination in the presence of high concentrations (3–6%) of exogenous glucose (Jang et al., 1997; Xiao et al., 2000). The use of a high concentration of glucose to obtain sugar-sensing effects has raised concerns about the physiological relevance of these assays (Leon and Sheen, 2003; Rook and Bevan, 2003). Unlike what was observed in the Arabidopsis study, tomato plants expressing *AtHXK1* exhibited sugar-sensing effects when grown in soil under natural growth conditions independent of exogenous sugar (Dai et al., 1999). It has been hypothesized that due to the growth inhibition effects of *AtHXK1*, transgenic Arabidopsis plants with high levels of *AtHXK1* expression were discriminated against throughout the transformation selection procedure, in favor of plants with lower levels of *AtHXK1* expression. As a result, only transgenic plants with moderate expression of *AtHXK1* were selected and, therefore, a high level of exogenous sugar was required to obtain a sugar-sensing response. To examine this hypothesis, Arabidopsis plants were transformed with *AtHXK1* and poorly growing kanamycin-resistant transformants were isolated. Indeed, several independent new transformants with high levels of *AtHXK1* expression exhibited classical sugar-sensing effects independent of exogenous sugar, alleviating the concern about the physiological relevance of these assays (Kelly et al., 2012). These new transgenic lines, together with the tomato lines that express high levels of *AtHXK1*, provide a way to study the role of *AtHXK1* at all developmental stages and in all plant organs and tissues.

The growth-arrest phenotype of Arabidopsis seedlings observed in the presence of exogenous sugars enabled the isolation of a number of sugar-insensitive and sugar-hypersensitive mutants. Characterization of these mutants revealed connections between sugar and plant hormone signaling pathways (reviewed in Leon and Sheen, 2003; Rolland et al., 2006; Rognoni et al., 2007; Ramon et al., 2008). Several of the isolated mutants turned out to be allelic to known ABA-synthesis (*aba*) and ABA-insensitive (*abi*) mutants (Zhou et al., 1998; Arenas-Huertero et al., 2000; Laby et al., 2000; Rook et al., 2001; Cheng et al., 2002). Overexpression of *AtHXK1* in the glucose insensitive (*gin*) mutants *gin1/aba2* and *gin5/abi4* does not restore the glucose sensitivity, indicating that ABA acts downstream of *AtHXK1* and is required for *AtHXK1*-mediated glucose responses (Zhou et al., 1998; Arenas-Huertero et al., 2000). In addition, exogenous glucose increased the expression of ABA-synthesis and signaling genes, as well as endogenous ABA levels (Cheng et al., 2002). It has been concluded that ABA plays a central role in *AtHXK1*-mediated sugar-signaling effects (Leon and Sheen, 2003). Yet, it is not known whether ABA biosynthesis is directly involved in the sugar signal transduction cascade or indirectly stimulated by sugars, modulating sugar-responsiveness (Ramon et al., 2008).

Unlike ABA, ethylene was shown to act in an antagonistic manner to glucose responses. The ethylene precursor 1-aminocyclopropane-1-carboxylic acid (ACC) prevented inhibition of cotyledon greening and seedling development at high concentrations of glucose in wild type seedlings (Zhou et al., 1998; Gibson et al., 2001). Repression by ACC of the glucose-dependent developmental arrest requires the AtHKL1 protein (Karve et al., 2012). A glucose insensitive (*gin*) phenotype was also displayed in constitutive ethylene biosynthesis (*eto1*) and constitutive ethylene signaling (*ctr1*) mutants (Zhou et al., 1998; Leon and Sheen, 2003; Moore et al., 2003; Rolland et al., 2006; Rognoni et al., 2007; Ramon et al., 2008; Cho et al., 2010). Lastly, the ethylene-insensitive mutants *etr1-1*, *ein2*, *ein3* as well as *mkk9* exhibit glucose hypersensitivity (Ramon et al., 2008). Thus, ethylene acts as an antagonist of the glucose response while ABA promotes it. A link between these hormones with respect to *AtHXK1*-related sugar effects has been studied with the double mutants *gin1/aba2 etr1* and *gin1/aba2 ein2*. These double mutants display the glucose insensitive phenotype of the *gin1/aba2* mutant (Zhou et al., 1998; Yanagisawa et al., 2003). Hence, ABA appears to be epistatic over ethylene, which seems to affect glucose response through ABA. While the connection between ABA, ethylene and *AtHXK1* has been observed in Arabidopsis seedlings grown in the presence of high concentrations (6%) of glucose, that connection was uncoupled at low (2%) concentration of glucose in absence of nitrogen source (Cho et al., 2010). Therefore, it has been suggested that early seedling developmental arrest in glucose is mediated by *AtHXK1* independent of ABA and ethylene.

The molecular mechanism of the *AtHXK1*-mediated sugar-sensing is not known. It has been demonstrated that a small fraction of the mitochondria-associated AtHXK1 is transported to the nucleus, where it forms a complex that includes the vacuolar H^+^-ATPase B1(VHA-B1) and the 19S regulatory particle of the proteasome subunit (RPT5B) (Cho et al., 2006b). This complex can bind the promoters of specific genes, such as *CAB2*, and may modulate their expression independent of glucose metabolism. It is likely that a conserved glucose binding site on AtHXK1 acts as a sensor and responds directly to the presence of glucose. It has been suggested that glucose-induced conformation change of AtHXK1 could alter the activity of VHA-B1 and RPT5B in the putative nuclear sugar-sensing complex (Cho et al., 2006b). Whether glucose promotes the transport of AtHXK1 to the nucleus or facilitates the complex formation has not been examined.

In addition to the original sugar-sensing roles of *AtHXK1* (e.g., regulating seedling development, photosynthesis, and plant growth), *AtHXK1* accelerates senescence, enhances the appearance of lateral buds, affects root growth, closes stomata, and controls transpiration (Dai et al., 1999; Xiao et al., 2000; Kelly et al., 2012). These effects were observed when *AtHXK1* was expressed under the global promoter 35S; whether native expression levels of *AtHXK1* in various plants parts and tissues also regulate these physiological responses remains to be addressed. The *AtHXK1* mutant *gin2* exhibited a smaller root system, tiny leaves and delayed senescence, but this phenotype may indicate a metabolic role for *AtHXK1* (Moore et al., 2003). Yet, transgenic Arabidopsis plants expressing the two catalytically inactive *AtHXK1* mutant alleles (G104D and S177A) in the *gin2* null mutant background displayed substantial leaf expansion when grown in soil in the presence of intense light. Therefore, it has been suggested that catalytically inactive *AtHXK1* mutants support both growth-inhibiting and growth-promoting roles of *AtHXK1* under different growth conditions (Moore et al., 2003).

The effect of HXK on Arabidopsis seedlings growing on high-sugar-containing medium (6% glucose) was showed to be dependent on auxin as well (Moore et al., 2003). However, these results differ from those presented of recent work performed with mature Arabidopsis plants grown in soil, in which high levels of *AtHXK1* expression suppressed auxin-response genes (Kelly et al., 2012). These opposite effects might be due to the different developmental stages examined in the two experiments.

A role for HXK in sugar-sensing has been reported not only for *AtHXK1*, but also for other mitochondria-associated (type B) HXKs. Expression of potato *StHXK1* and *StHXK2* and rice *OsHXK5* and *OsHXK6* in Arabidopsis plants lacking *AtHXK1* (*gin2* mutant) complemented glucose sensitivity, indicating their putative role in sugar-sensing (Veramendi et al., 2002; Cho et al., 2009a,b). Transgenic rice plants overexpressing *OsHXK5* or *OsHXK6* exhibited growth inhibition and reduced expression of photosynthetic genes in response to glucose treatment (Cho et al., 2009a). In addition, sense and antisense expression of the Arabidopsis *AtHXK2* in Arabidopsis plants, as well as the expression of poplar (*Populus trichocarpa*) *PtHXK1* and rice *OsHXK5*, *OsHXK6* in plant protoplasts were also correlated with photosynthetic gene expression, suggesting that these enzymes play a sensing role as well (Jang and Sheen, 1997; Cho et al., 2009a; Karve et al., 2010). Recent work has suggested that the mitochondria-associated non-catalytic homolog of AtHXK1, HKL1, may also mediate some glucose responses in Arabidopsis (Karve and Moore, 2009; Karve et al., 2012). A screen of HXKs from *Selaginella* revealed that a cytosolic HXK, *SmHXK3*, also conveys repression of a photosynthetic gene in maize protoplasts (Karve et al., 2010). This is the first evidence of a non-mitochondria-associated HXK that is involved in sugar-sensing. It would be interesting to test whether other cytosolic HXKs in monocots and mosses play similar roles.

The physiological role of the plastidic (type A) HXKs is not yet known, but was examined in a single study of the Arabidopsis plastidic HXK *AtHXK3*. In that study, the *AtHXK3* knockout mutant was found to be insensitive to 7% glucose, suggesting that type A HXKs may also play a role in sugar-sensing (Zhang et al., 2010). In *Physcomitrella*, the plastidic *PpHXK1* was found to regulate development by controlling the type of filamentous gametophyte formed. Mosses have two types of filaments, chloronemata cells, which are photosynthetically active, and caulonemata cells, which spread the colony. Using a knockout *hxk1* mutant, Olsson et al. (2003) and Thelander et al. (2005) demonstrated that when there is a surplus of available energy, there is a shift from the production of chloronemata to the production of caulonemata. This shift is reversed when the energy supply is limited. This cell-type transition is mediated by the plastidic *PpHXK1* (Olsson et al., 2003; Thelander et al., 2005).

It has also been suggested that HXKs may prevent programmed cell death (Kim et al., 2006), dictate actin-filament reorganization (Balasubramanian et al., 2007, 2008), and regulate seed development (Troncoso-Ponce et al., 2011), starch content (Veramendi et al., 1999; Giese et al., 2005), and pollen germination (Xu et al., 2008). HXKs may also be involved in biotic and abiotic stress responses (Claeyssen and Rivoal, 2007; Sarowar et al., 2008), particularly responses to pathogens (Sarowar et al., 2008). A study performed in wheat demonstrated that HXK can also control triose-phosphate/phosphate translocator content, thereby affecting the distribution of assimilates in the chloroplasts (Sun et al., 2006). Yet the question of which of the above roles are related to sugar-sensing and which are the result of the metabolic catalytic functions of HXK remains to be studied.

### Role of FRK

Compared to the roles of HXK, the physiological roles of the different FRK isozymes in plants are less clear. The lack of FRK plant mutants may suggest that FRK genes are either essential or have highly redundant functions under normal growth conditions. Consequently, information about the function of FRK isozymes has been gathered mostly from transgenic plants and through the examination of gene expression profiles under different growth conditions. It has been proposed that FRKs affect starch accumulation in different plant species, including tomato (Schaffer and Petreikov, 1997b). Yet, analysis of *FRK2*- and *FRK1*-antisense tomato and potato plants demonstrated that starch accumulation is not affected by *FRK* (Dai et al., 2002a; Odanaka et al., 2002; Davies et al., 2005). Instead, *FRK2* was found to be essential for vascular development (German et al., 2003; Damari-Weissler et al., 2009). Reduced expression of *FRK2* in antisense plants resulted in deformed vasculature, smaller cell size in the xylem and phloem, reduced cambium activity and secondary walls in vessels, and small sieve elements with low levels of callose deposition (Damari-Weissler et al., 2009). The development of xylem and phloem is dependent upon sucrose metabolism in the vascular system (Figure 1). To be metabolized, sucrose must first be cleaved by either INV or sucrose synthase (SUS). *SUS1*, *SUS2*, and *FRK2* are expressed at high levels in the vascular tissues of tomato stems (German et al., 2003; Goren et al., 2011). Both products of sucrose cleavage by SUS, UDP-G, and fructose, might be central for vascular development. UDP-G may be used for cellulose and cell wall synthesis, while phosphorylated fructose can be utilized for energy production or fed into other metabolic pathways. SUS is feedback-inhibited by its product, fructose, when the concentration of fructose exceeds 0.5–1 mM (Schaffer and Petreikov, 1997a) (Figure 1). Hence, the phosphorylation of fructose by *FRK2* might be necessary for the sucrose cleavage, sugar metabolism, and cell wall synthesis that are essential for proper development of the vascular tissues (German et al., 2003; Damari-Weissler et al., 2009).

Reduced FRK activity in potato due to antisense suppression of *StFK1* (the potato homolog of tomato *FRK2*) resulted in reduced tuber yield (Davies et al., 2005). Although that report did not include a detailed analysis of the vasculature system of the affected plants, it is tempting to speculate that deformation of the vascular system is responsible for the reduced tuber yield. The involvement of *FRK* in the vasculature of potato tubers is further supported by the results of *in situ* staining of *FRK* activity showing localization in vascular bundles (Sergeeva and Vreugdenhil, 2002).

A physiological role for *FRK1* has been suggested based on the phenotype of *FRK1*-antisense tomato plants. Although both *FRK1*- and *FRK2*-antisense transgenic tomato plants exhibited reduced carbohydrate content, the transition to flowering was delayed only in the *FRK1*-suppressed plants (Odanaka et al., 2002). Sugar involvement in flowering transition has been reported for Arabidopsis and tobacco. In those species, impaired sugar translocation resulted in delayed flowering while an increase in sugar synthesis caused plants to flower early (Burkle et al., 1998; Corbesier et al., 1998; Heyer et al., 2004). Although the mechanism by which carbohydrates control the transition to flowering is not yet clear, the different phenotypes of antisense *FRK1* and *FRK2* suggest that the transition to flowering is not simply affected by carbohydrate status, but rather that *FRK1* might mediate a signal promoting this process. Still, an additional line of evidence is required to support this hypothesis.

Fructokinases has also been shown to play a role in anther development. The developing anther is a photosynthetically inactive organ and thus requires sucrose as a source of energy for its development. It has been suggested that FRK plays a central role in providing fructose 6-phosphate and thus facilitating the production of UDP-G to support the synthesis of cellulose for the elongating cell wall (Karni and Aloni, 2002). A recent study suggested a role for FRK in providing F6P for sucrose synthesis (Pressman et al., 2012). The further biological significance of FRK has been demonstrated by the specific expression of *FRK4* in tomato anthers during late stages of pollen development and during pollen germination (German et al., 2002). Interestingly, the Arabidopsis FRK ortholog (At4g10260) displays a similar restricted expression pattern. It is likely that other species possess pollen-specific FRK isozymes as well (David-Schwartz et al., 2013). In a study that aimed to elucidate the protein interaction network underlying the process of pollen development in rice, Kerim et al. (2003) found two isoforms of FRKII that were up-regulated in late stages of pollen development. These isoforms were co-regulated with two isoforms of vacuolar acid INV. The up-regulation of both INV and FRK coincides with increased starch accumulation in the developing pollen grains (Kerim et al., 2003). Hence, FRK may be implicated in both the accumulation of starch during pollen development and the degradation of starch during pollen tube elongation.

The involvement of FRK in plant responses to abiotic stress has been reported recently in sunflower, maize, and rice. In sunflower, proteins related to basic carbon metabolism, including an ortholog of the plastidic *SlFRK3*, are up-regulated in response to drought stress (Fulda et al., 2011). Up-regulation of *OsFK2* was reported in rice under anoxic conditions, implying that this gene plays a role in anaerobic energy production. In contrast, *OsFK1* is expressed under aerobic conditions (Guglielminetti et al., 2006). Another example of a specific FRK isozyme that is expressed in response to abiotic stress was found in maize, in which FRK2 is up-regulated in response to short-term salt stress (Zorb et al., 2011). This isozyme, together with other carbohydrate-metabolism enzymes, may serve as a marker for early signs of salt stress (Zorb et al., 2011). The expression of specific FRK isozymes in response to abiotic stress may imply a role for this enzyme in plants’ adaptations to various types of stress.

## Summary and Avenues for Future Work

The phosphorylation of glucose and fructose by HXK and FRK is pivotal for all metabolic processes. Most plant species have a single plastidic HXK and a single plastidic FRK, with multiple HXK and FRK isozymes in the cytoplasm. While the cytoplasmic FRKs are located within the cytosol, all of the cytoplasmic HXKs in eudicots and most of the cytoplasmic HXKs in monocots are associated with the mitochondria. HXKs may also appear in the nucleus. The different intracellular locations of HXKs and FRKs are of particular interest. They not only support the theory that HXKs and FRKs play different roles, but also raise questions about the intracellular trafficking of glucose and fructose.

Sugar-sensing roles (in addition to the metabolic function) with regard to several physiological processes, primarily related to photosynthesis and photosynthetic tissues, have been documented for a few HXKs. However, the molecular mechanisms of these processes and the roles of the various HXKs in sink and other tissues are not yet understood. Specifically, the molecular and physiological nature of the interactions between HXKs and plant hormones remain unclear. Unlike some of the HXKs, there is no strong evidence that FRK plays any sugar-sensing role. Rather, FRK may function primarily in the regulation of sugar metabolism in sink and vascular tissues. Nevertheless, the roles of HXK and FRK may be dictated by the specific tissues and cell types in which they are found. Therefore, it is essential to explore in which tissues and types of cells the various HXK and FRK genes are expressed. Later on, tissue-specific modulated expression of the corresponding genes (using mutants or gene expression under specific promoters) may help clarify the roles of these enzymes in different tissues.

The study of both HXK and FRK has led to significant discoveries, such as the dual-function of the mitochondria-associated *AtHXK1* and the role of FRK in vascular development. Due to the central roles of HXK and FRK in plant physiology, it is very likely that the modulation of specific HXK or FRK isozymes in certain tissues may have profound effects on specific economic traits. For example, FRK may control the amount of sugar allocated for vascular tissues and may be used to enhance xylem and vascular development in woody plants. Similarly, HXK may be used to control transpiration in agricultural crops. We believe that these discoveries and the potential uses of these enzymes will encourage further exploration of these gene families.

## Conflict of Interest Statement

The authors declare that the research was conducted in the absence of any commercial or financial relationships that could be construed as a potential conflict of interest.
